# Geographical distribution of the giant honey bee *Apislaboriosa* Smith, 1871 (Hymenoptera, Apidae)

**DOI:** 10.3897/zookeys.951.49855

**Published:** 2020-07-22

**Authors:** Nyaton Kitnya, M.V. Prabhudev, Chet Prasad Bhatta, Thai Hong Pham, Tshering Nidup, Karsing Megu, Jharna Chakravorty, Axel Brockmann, G.W. Otis

**Affiliations:** 1 Department of Zoology, Rajiv Gandhi University, University Road, Itanagar, Papum Pare, Arunachal Pradesh 791112, India Rajiv Gandhi University Itanagar India; 2 National Centre for Biological Sciences - Tata Institute of Fundamental Research, Bellary Road, Bangalore 560065, Karnataka, India Tata Institute of Fundamental Research Bangalore India; 3 Department of Biosciences, University of Mysore, Krishnaraja Boulevard Road, K.G. Koppal, Mysore 570006, Karnataka, India University of Mysore Koppal India; 4 Department of Ecology and Evolutionary Biology, University of Kansas, 1200 Sunnyside Avenue, Lawrence, KS 66045, USA University of Kansas Lawrence United States of America; 5 Department of Biology, Radford University Carilion, 101 Elm Avenue SE, Roanoke, VA 24013, USA Radford University Carilion Roanoke United States of America; 6 Research Center for Tropical Bees and Beekeeping, Vietnam National University of Agriculture, Trau Quy - Gia Lam - Ha Noi, Vietnam Vietnam National University of Agriculture Ha Noi Vietnam; 7 Department of Environment & Life Sciences, Sherubtse College, Royal University of Bhutan, Kanglung, Trashingang, Bhutan Royal University of Bhutan Trashingang Bhutan; 8 School of Environmental Sciences, University of Guelph, Guelph, Ontario N1G 2W1, Canada University of Guelph Guelph Canada

**Keywords:** Apidae, *Apisdorsata*, conservation, Himalayas, pollinator, sympatry

## Abstract

Worldwide pollinator declines have dramatically increased our need to survey and monitor pollinator distributions and abundances. The giant honey bee, *Apislaboriosa*, is one of the important pollinators at higher altitudes of the Himalayas. This species has a restricted distribution along the Himalayas and neighbouring mountain ranges of Asia. Previous assessments of its distribution, published more than 20 years ago, were based on museum specimens. Since then, 244 additional localities have been revealed through field trips by the authors, publications, and websites. We present a revised distribution for *A.laboriosa* that better defines its range and extends it eastward to the mountains of northern Vietnam, southward along the Arakan Mountains to west-central Myanmar, into the Shillong Hills of Meghalaya, India, and northwestward in Uttarakhand, India. This species is generally found at elevations between 1000–3000 m a.s.l.. In northeastern India *A.laboriosa* colonies occur during summer at sites as low as 850 m a.s.l. and some lower elevation colonies maintain their nests throughout the winter. Finally, we report three regions in Arunachal Pradesh, India, and nine locations in northern Vietnam, where we observed workers of *A.laboriosa* and *A.dorsata* foraging sympatrically; their co-occurrence supports the species status of *Apislaboriosa*.

## Introduction

The Himalayan giant honey bee *A.laboriosa* is a spectacular but poorly understood species, in large part because it usually nests on inaccessible cliff faces in the Himalaya Mountains (Cronin 1979; Sakagami et al. 1980; Roubik et al. 1985; Underwood 1990a; Joshi et al. 2004; Gogoi et al. 2017). The first specimen was collected in the mountainous regions of western Yunnan and named by Frederick Smith (Moore et al. 1871), who noted several characteristics that he felt distinguish *A.laboriosa* from lowland *A.dorsata*. This taxon was subsequently ignored until Maa (1953) undertook his reassessment of honey bee taxonomy. He stated its distribution as “India (Sikkim; Assam); China (western Yunnan). Probably also occurring in N. Burma”. Sakagami et al. (1980) provided the first detailed descriptions of the morphology, biology, and geography of *A.laboriosa* and provided strong evidence that it should be recognized as a distinct species different from the lowland giant honey bee *A.dorsata*. They also presented a range map depicting 22 localities along several rivers that extend into the Himalayas of Nepal. They noted that records outside Nepal were “scarce” and provided just one locality in “Tibet” and four in Arunachal Pradesh (Sakagami et al. 1980: p. 63). Interestingly, Sakagami et al. (1980) mentioned one site (Denling Forest, Kameng Div., Arunachal Pradesh, 229 m a.s.l.) from which both *A.laboriosa* and *A.dorsata* had been collected, indicating that in some parts of Asia these two forms occur sympatrically.

The most recent range map of *A.laboriosa* (Otis 1996) was published more than 20 years ago. Although Otis (1996) compiled all locality data available at that time, there were obvious gaps (e.g., in Bhutan, northeastern India, northern Myanmar, Laos, and Vietnam). Since then, the number of verified reports has increased rapidly due to additional fieldwork, new publications and postings of photos and videos by naturalists on iNaturalist and other websites.

We present here an updated distribution map of *A.laboriosa* and present additional evidence that *A.laboriosa* and its sister species *A.dorsata* co-occur in several locations in Asia.

## Materials and methods

### Identification of *Apislaboriosa*

**Figure 1.**
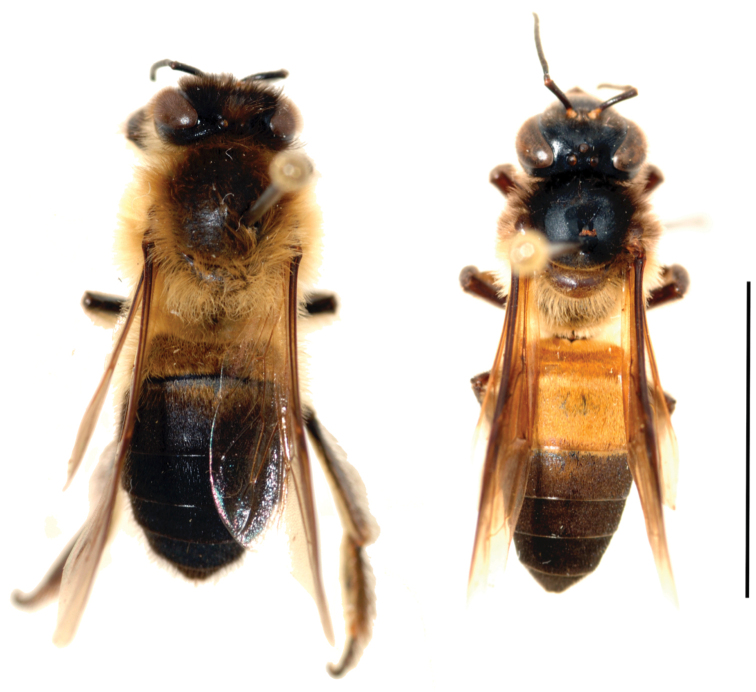
*Apislaboriosa* and *Apisdorsata* worker bees. *A.laboriosa* (left) has a completely dark abdomen and long golden thoracic hairs. *A.dorsata* (right) has several orange or yellow anterior abdominal segments and dark thoracic hairs. Details for the specimens photographed: *A.laboriosa*, collected by BA Underwood, Kaski District, Nepal, 1860 m, 8 v 1984 (Nest 6–8); *A.dorsata*, collected by GW Otis, Serdang, Selangor, Malaysia, 3.00 N, 101.68 E, 8 ii 1989. Scale bar: 1 cm.

The Himalayan giant honey bee *A.laboriosa* differs significantly from the giant honey bee *A.dorsata* of mainland Asia in many characters noted by Sakagami et al. (1980). Sakagami et al. (1980) could always distinguish the two taxa on the basis of thoracic hair colour, which was “tawny yellow” in *laboriosa* and “mostly dark” in *dorsata*. Additionally, the first two gastral tergites of *laboriosa* are black (grey in callow adults); in *dorsata* of mainland Asia, they are orange-brown (pale yellow in callows; Fig. 1). Several genetic analyses indicate these two taxa have diverged sufficiently to consider *A.laboriosa* to be a distinct species (Arias and Sheppard 2005; Raffiudin and Crozier 2007; Lo et al. 2010; Chhakchhuak et al. 2016; Takahashi et al. 2018). Despite these many differences, Engel (1999), in the most recent reassessment of *Apis* taxonomy, considered *A.laboriosa* to be a subspecies of *A.dorsata* that may be deserving of species status. Because of substantive differences in drone morphology (NK and GWO, unpubl. data) and distinct morphometric differences (NK, unpublished data) between the two taxa collected sympatrically in northeastern India (this study), we are confident that *A.laboriosa* is a distinct species and refer to it as such below. For this study, we identified specimens and photos of the bees on the basis of thoracic hair and abdominal colour (Sakagami et al. 1980).

### Data collection

The starting point for this project was the list of collection localities reported by Otis (1996) who summarized the 105 records available from museum specimens and literature up to that time (Maa 1953; Sakagami et al. 1980; Kuang and Li 1985; Roubik et al. 1985; Underwood 1990a; Batra 1996).

Subsequently, Trung et al. (1996) remarkably extended the distribution of *A.laboriosa* well into Vietnam. Additional localities in Vietnam have been detected in seven northern provinces by THP. Between 2001–2008 he sought information on cliff-nesting honey bees at high elevations (> 900 m a.s.l.) in northern Vietnam from beekeepers, honey-hunters, and others living in those regions. For those who responded positively, he visited the region and showed informants photos and specimens of *A.laboriosa* and *A.dorsata*. If the information suggested the bees were *A.laboriosa*, then he contacted them to visit the nesting cliff to observe the colonies and collect specimens, if possible, for verification.

CPB collected specimens from many parts of Nepal to analyze genetic variability within *A.laboriosa*; he contributed 17 additional localities in Nepal. Joshi et al. (2004) reported numerous localities (*N* = 54) in the Kaski District of Nepal.

TN contributed 29 new localities in Bhutan in addition to the two localities he and his colleague had reported earlier (Nidup and Dorji 2016).

Within India, a team (NK, MVP, KM, JC, and AB), in their fieldwork on *A.laboriosa*, have added numerous localities from northern and northeastern India. Several authors have reported sites where this bee species occurs in Uttarakhand, India (Gupta 2004; Joshi et al. 2008; Joshi et al. 2016). Additional localities were reported from Arunachal Pradesh, India, by Gogoi and colleagues (Gogoi et al. 2017; Tayeng and Gogoi 2018). The Nagaland Beekeeping and Honey Mission (NBHM) has collated information on the “Rock bee” within the state of Nagaland (Team NBHM 2015). Similarly, Chhakchhuak et al. (2016) reported a nesting cliff in Murlen in Mizoram. Vivek Sarkar (pers. comm.) shared three localities in the highlands of Meghalaya.

Hliang Min Oo (pers. comm.) located cliffs inhabited by ~50 colonies of *A.laboriosa* in the mountains of western Myanmar. He shared photos that are clearly of that species. Anne Schooffs (pers. comm.) and Kevin Kamp (pers. comm.) contributed their observations in Laos. Similarly, Xin Zhou and Li Fei Qui (pers. comm.) shared details of their 2019 collection locality in Yunnan, China. Cao and colleagues already reported two localities in Yunnan in 2012 (Cao et al. 2012).

We recognize that some records are stronger than others. Consequently, we have distinguished four categories of records:

1. Observations by the authors. These include observations of nests on rock cliffs and of foragers (many of which were collected for further study) at flowers and human urine.
2. Internet records. GWO searched the internet extensively for photographs of bees and bee nests that could be definitively identified as A. laboriosa based on abdominal and thoracic hair colour. This search included websites and images retrieved from Google searches of “ Apis laboriosa”, “cliff bee”, “giant honey bee + (country name)”, and “honeyhunting + (country name)”, with the search including all mountainous countries in Asia from Pakistan east to Vietnam; records of A. laboriosa posted to iNaturalist (www.inaturalist.org), all of which were verified as A. laboriosa by John Ashcher, National University of Singapore; and the Nature Picture Library (www.naturepl.com). When details were lacking in the post (locality, date, etc.), the people who had posted the sightings were contacted directly.
3. Records from published reports. Scientists who mentioned A. laboriosa in their works were presumably aware of the differences between A. laboriosa and A. dorsata. We trusted their identifications, i.e., in most cases we did not attempt to contact them to verify their ability to distinguish A. laboriosa.
4. Personal observations of naturalists and honey-hunters that cannot be verified by other means. Honey-hunters, through their extensive experiences, generally distinguish between A. laboriosa (the “cliff bee” with a black abdomen) and A. dorsata (the “tree bee” with the orange abdomen). In cases where they differentiated these two forms, we included the localities they reported. We have used different coloured symbols for these different categories of information on our revised distribution map, with category 1 records being the uppermost layer on the map. Some lower category localities are obscured by stronger higher category records.

We obtained additional bee localities by searching for photos on the internet and literature about the yellow-rumped honeyguide (*Indicatorxanthonotus*). The honeyguides (Indicatoridae) are one of the few taxa of animals known to be able to digest wax (Downs et al. 2002). Males of this Himalayan species defend empty combs of *A.laboriosa*, then mate with females that arrive to feed on beeswax (Cronin and Sherman 1976; Cronin 1979; Underwood 1992). The geographic and elevational distributions of the yellow-rumped honeyguide (Bird Life International; www.birdlife.org) closely match those of *A.laboriosa* (this study). We performed Google and iNaturalist searches for “yellow-rumped honeyguide” and “*Indicatorxanthonotus*”. Localities for the honeyguide at elevations >1500 m a.s.l. that included depictions or descriptions of them associated with open-nesting honey bees have been included.

In our personal field work, we used global positioning system (GPS) instruments (i.e., eTrex 20, Garmin Ltd, Olathe, Kansas, USA) to document locations. When that was not possible or we had only a locality name (e.g., many of the localities reported in publications), we searched for them using Google Maps. Occasionally due to changes in names or spellings, we undertook lengthy web searches to find the current names. Not all records could be located (e.g., “Pamir, Arunachal Pradesh” reported by Sakagami et al. 1980). Latitude and longitude coordinates are presented in degrees and decimal degrees (Suppl. material 1: Table S1).

We plotted locality records using ArcGIS Desktop 10.3 of ESRI (Environmental System Research Institute, www.esri.com). The boundary maps of the region of interest were extracted from Google Earth Pro (v7.3) as a Keyhole Markup language Zipped (kmz) file and imported into ArcGIS.

## Results

### Revised distribution of *Apislaboriosa*

**Figure 2.**
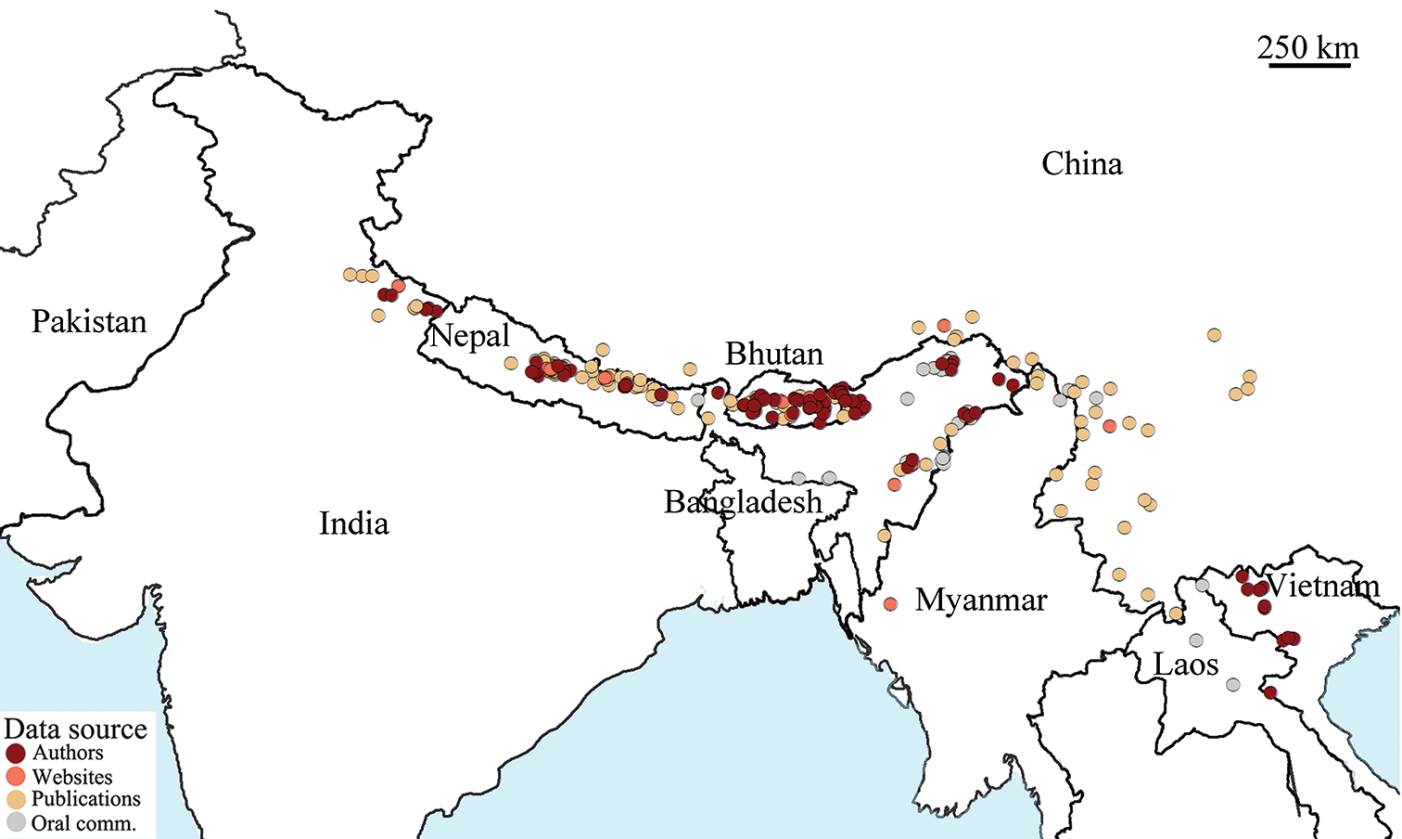
Geographical distribution of *Apislaboriosa*. Each circle indicates a locality at which a nest of *A.laboriosa* or workers foraging on flowers were found. The color indicates the source of information. Dark red: information collected by one or several of the authors; orange: photos published on websites; tan: information from published papers; and grey: oral reports by colleagues or local people. Scale bar: 250 km.

We have compiled a list of 349 localities of *Apislaboriosa* foragers or nests (Suppl. material 1), of which we were able to locate and map 345 (see Fig. 2). The mapped localities are as follows: Bhutan (57), China (48), India (92), Laos (3), Myanmar (4), Nepal (132), and Vietnam (13). The species is distributed almost continuously over a distance of >2500 km along the Pan-Himalaya region from Uttarakhand, India, eastward through Nepal, Sikkim and northern West Bengal (Darjeeling), Bhutan, northeastern India, Yunnan and southern Tibet in China, and the northern portions of Myanmar, Laos, and Vietnam. We report for the first time numerous records southward along the Arakan Mountains in eastern Arunachal Pradesh, Nagaland, Manipur, and Mizoram (India) to Matupi in west-central Myanmar. We have also verified that it occurs in the Shillong Hills of Meghalaya.

### Elevational distribution of *Apislaboriosa*

**Figure 3.**
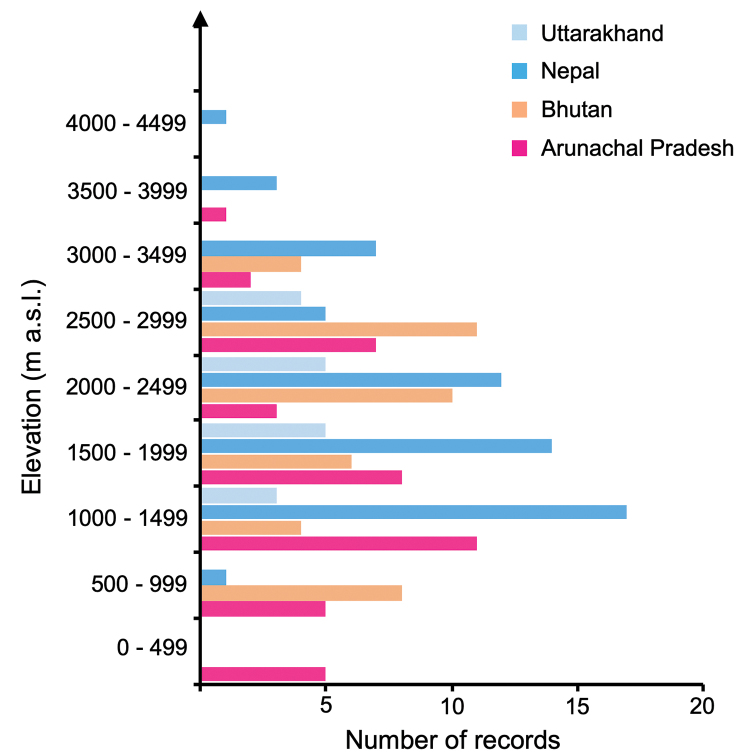
Elevational distribution of *A.laboriosa* records for Uttarakhand, Nepal, Bhutan and Arunachal Pradesh. 94% of all records were found between 500–3500 m a.s.l.. The lowest occurrence of *A.laboriosa* was observed in Arunachal Pradesh (229 m a.s.l.), and the highest in Nepal (4267 m a.s.l.). Uttarakhand (*N* = 17; range: 1008–2743 m a.s.l.; mean: 1927 ±131 m), Nepal (*N*= 60; range: 800–4100 m a.s.l.; mean: 2036 ±103 m), Bhutan (*N* = 43; range: 631–3399 m a.s.l.; mean: 2077 ±124 m), Arunachal Pradesh (*N* = 17; range: 229–3649 m a.s.l.; mean: 1620 ±143 m).

Localities range in elevation from approximately 230–4270 m a.s.l. Nearly all records (94%) fall within the altitudinal range of 500–3500 m a.s.l., and 77.2% were between 1000–3000 m a.s.l. (Fig. 3). We confirmed the observation of Sakagami et al. (1980) that *A.laboriosa* occurs at lower elevations in Arunachal Pradesh than at sites further west in Bhutan, Nepal, and Uttarakhand, India. The lowest recorded elevation was reported by Sakagami et al. (1980), at 229 m a.s.l. in “Denling Forest”, western Arunachal Pradesh, India. We were unable to find that locality to map it. However, we (NK, KM, and GWO) observed foragers at a similar elevation (233 m a.s.l.) in central Arunachal Pradesh. Relatively few records are from sites higher than 3000 m a.s.l. and the records from Trubuking Kharka, Nepal (4100 m a.s.l.; Sakagami et.al.1980) and a specimen in the Natural History Museum, London (4267 m a.s.l.) are the only observations above 4000 m a.s.l.

### Sympatric occurrence of *Apislaboriosa* with *Apisdorsata*

**Figure 4.**
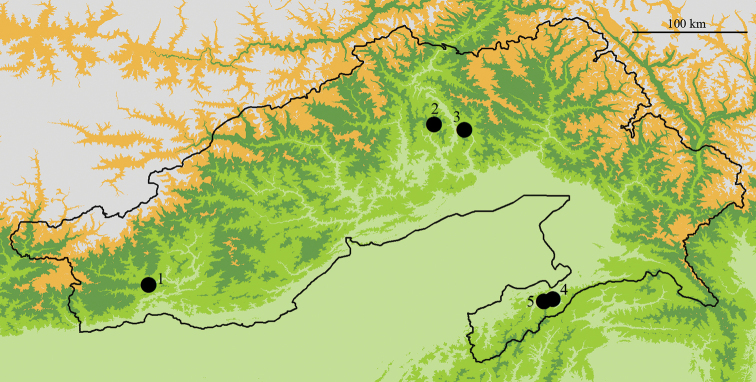
Sites of sympatric occurrence of *Apislaboriosa* and *Apisdorsata* in Arunachal Pradesh, India. All five localities (black dots) where we found *A.laboriosa* and *A.dorsata* foraging together were below 1500 m a.s.l. (1) West Kameng District, Nag Mandir, 27.203N, 92.561E, 1164 m a.s.l; (2) West Siang District, Tumbin, 28.456N, 94.684E, 356 m a.s.l; (3) Siang District, Modi, 28.487N, 95.087E, 534 m a.s.l; (4) Tirap District, Kala Pahar, 26.934N, 95.576E ,1470 m a.s.l; (5) Tutnyu, 26.962N, 95.631E, 1060 m a.s.l. Scale bar: 100 km.

In their field trips, the Indian team discovered five sites in three regions of Arunachal Pradesh in northeastern India where *A.laboriosa* foraged together with its sister species *A.dorsata*. The regions and sites were: (1) Western Arunachal: West Kameng District, Nag Mandir; (2) Central Arunachal: West Siang District, Tumbin and Siang District, Modi; and (3) Southeast Arunachal: Tirap District, Kala Pahar and Tutnyu (Fig. 4). Additionally, THP observed both species in close proximity at nine sites in northern Vietnam. These are located in five provinces: Hoa Binh, Lao Cai, Lai Chau, Son La, and Yen Bai. The details of these records are presented in Suppl. material 1.

## Discussion

*Apislaboriosa* inhabits a 2500 km swath along the southern edge of the Pan-Himalaya region. We have added considerably to the distribution of this species as last presented by Otis (1996). First, we have added many additional localities for this species in Uttarakhand in northern India, the eastern portion of Nepal, all of Bhutan, and much of Arunachal Pradesh in northeastern India, demonstrating that this species is widespread over that region. More importantly, the records we have compiled show range extensions eastward to several provinces in northern Vietnam (first reported there by Trung et al. 1996) and southward for 600 km in the Arakan Mountains (Patkai Range, Naga Hills, and Mizo Hills of Nagaland, Manipur, and Mizoram) to 21.7N latitude in the Chin Hills of Myanmar. We also report for the first time *A.laboriosa* from the Shillong Plateau in Meghalaya, India.

Very few (*N* = 10; 6.2%) collections and observations have been made at locations situated at elevations below 500 m or above 3500 m a.s.l. (Fig. 3). Roubik et al. (1985) reported the mean elevations of their observations in central Nepal to be 3143 m a.s.l. (range from 1800–3800 m a.s.l), which is much higher than the mean of 2036 m a.s.l. (range from 800–4100 m a.s.l.) determined from our more complete data set. Our new observations have confirmed the initial observation of Sakagami et al. (1980) that this species occurs at lower elevations in northeastern India than in Nepal (Fig. 3). However, simply mapping localities of nests and foraging bees is static and fails to recognize the dynamic elevational migrations of colonies. Underwood (1990a) reported that *A.laboriosa* colonies abandon most nesting cliffs in fall and spend the winter in combless swarms at lower elevations. By April they have generally recolonized lower elevation nesting cliffs. As the season progresses, colonies continue to move higher in elevations along river valleys during the summer, before they retreat downhill for the winter. The records we have reported (Suppl. material 1) lack sufficient elevational and temporal details to reconstruct patterns of seasonal migrations. However, preliminary observations by NK in Arunachal Pradesh suggest that both *A.laboriosa* and *A.dorsata* migrate considerable distances along river valleys leading into the mountains during summer months. Detailed observations throughout the seasons along elevational transects are warranted.

Earlier research reported that the altitudinal ranges of *A.laboriosa* and its sister species *A.dorsata* differ substantially (Sakagami et al. 1980; Roubik et al. 1985; Oldroyd and Wongsiri 2006; Hepburn and Radloff 2011). However, Sakagami et al. (1980) commented that these species were both collected at the Denling Forest in Kameng Division, West Arunachal, at an elevation 229 m a.s.l. on 5 May. This relatively late date in spring suggests these bees were nesting near that site. We have confirmed the sympatric occurrence of these two species in five additional locations within three widely separated regions of Arunachal Pradesh (Fig. 3) and five provinces within northern Vietnam. All of these locations were at altitudes below 1500 m a.s.l. Interestingly, Joshi and colleagues (Joshi et al. 2008; Joshi et al. 2016) reported these two species as temporally occurring sympatrically at elevations of 2100–2800 m a.s.l. in Uttarakhand, India; and THP found them co-occurring in nine locations in northern Vietnam. Thus, depending on the environments, both species show variation in their altitudinal range (Underwood 1990a; Woyke et al. 2001; Joshi et al. 2008). More detailed collections and observations of *A.laboriosa* and *A.dorsata* at such sites will be required to verify whether these honey bees hybridize where they occur sympatrically (McEvoy and Underwood (1988). However, till today there is no evidence of intermediate forms where the two species co-occur (Sakagami et al. 1980; Roubik et al. 1985); rather, preliminary morphometric analyses of the specimens from the areas of sympatry in Arunachal Pradesh confirm their differences in size, shape, and colour (NK, unpublished data). If further collections confirm the preliminary conclusion that they maintain their distinctive species-specific characters in sympatry, that will provide additional support for the status of *A.laboriosa* as a distinct species.

Separate from its species status, *A.laboriosa* shows several unique characters that seem to be specific adaptations to living in mountainous habitats. Comparative studies of *A.laboriosa* and *A.dorsata* have shown that they differ in behaviour such as thermoregulation of thoracic temperature during flight (Underwood 1991); minimum temperature for foraging flight activity (Woyke et al. 2012); dorso-ventral abdomen flipping to stabilize body temperature (Woyke et al. 2008) and mating flight times, i.e., early afternoon in *A.laboriosa* (Underwood 1990b) compared to after sunset in *A.dorsata* (Tan et al. 1999; Otis et al. 2000). They also differ in other behaviours that may be related to living in high elevation, such as dance communication (Kirchner et al. 1996), pheromonal chemistry (Blum et al. 2000) and defensive body movements (Woyke et al. 2008).

*Apislaboriosa* is notably absent from the western third of Nepal, from 80.5N to 82.6E longitude. This may reflect the relatively dry climate of western Nepal, a lack of collections, or habitat degradation. Field work in several other mountainous portions of Asia may detect this species. These include:

1. northeastern Myanmar (Kumon Range and Gooligong Mountains), eastern Myanmar (much of Shan State), northern Laos (e.g., Annam Highlands and Xiangkhoang Plateau), and possibly extreme northern Thailand (e.g., Doi Pha Hom Pok National Park);
2. the valleys of the Mekong, Yangtze, Yalong, and Dadu rivers that extend into the southeastern edge of the Tibetan Plateau.
3. northeastern Punjab, Pakistan, and western Jammu, India.

Several lines of evidence point to the existence of *A.laboriosa* in Pakistan. Khan et al. (2014) reported several specimens of giant honey bees they collected in Murree, Pakistan (33.92N, 73.40E) at an elevation of ~2300 m a.s.l. as “*A.dorsata*”, despite the general understanding that *A.dorsata* lives below 1200 m a.s.l. elevation in Pakistan (Muzaffar and Ahmed 1990). Unfortunately, the specimens from that study were not retained. Historically, the yellow-rumped honeyguide (*Indicatorxanthonotus*), a bird with an intimate association with *A.laboriosa* combs (Cronin and Sherman 1976, Underwood 1992, Inskipp et al. 2008), has been observed in Murree (Magrath 1909). Just 60 km to the east of Murree, giant honey bees collected in Poonch, Jammu, India (33.82N, 74.12E), differed markedly in morphometric analyses from other *A.dorsata* specimens from Jammu and the rest of India (Mutharaman et al. 2013). Finding *Apislaboriosa* in this region would extend its distribution another 400–500 km northwestward.

## Conclusion

Worldwide pollinator declines have increased the urgency to survey abundances of pollinators and to study their biology and ecology for their conservation. Asian honey bees and in particular species like *A.laboriosa*, with a restricted distribution in areas difficult to access, are dramatically understudied. Our study provides a revised description of the distribution of the Himalayan giant honey bee, *Apislaboriosa*. This is a necessary step to revitalize studies on this important pollinator species in the Himalayas (Batra 1996).

Numerous reports on *A.laboriosa* indicate that this honey bee shows specific adaptations to living in high elevation mountainous areas compared to other more tropical honey bee species. Detailed studies on its biology promise to provide interesting insights into the evolutionary history and plasticity of honey bee physiology and social behavior. Locations where *A.laboriosa* and *A.dorsata* co-occur temporally, like those we report in Arunachal Pradesh and Vietnam, are particularly suitable regions for future studies.

## Supplementary materials

Supplementary material 1 Collection locality information Data type Excel spreadsheet Explanation note Abbreviations as follows – Type of data: Author’s observation (1), Photo from website (2), Publications (3), Personal communication (4). Source of Information: Authors’ initials- Chet Prasad Bhatta (CPB), Gard Williams Otis (GWO), Karsing Megu (KM), Nyaton Kitnya (NK), M.V. Prabhudev (MVP), Thai Hong Pham (THP), Tsering Nidup (TN); Organizations and Museums: Nagaland Beekeeping and Honey Mission (NBHM), Natural History Museum United Kingdom (NHMUK), National Museum of Natural History, US (USNM), Rijksmuseum of Natural History, Leiden, the Netherlands (RNH). Sakagami et al. (1980) and Underwood (1990a) numbered localities in their papers. Asterisks (*) indicate sites where *A.laboriosa* and *A.dorsata* occurred sympatrically. https://binary.pensoft.net/file/434688This dataset is made available under the Open Database License (http://opendatacommons.org/licenses/odbl/1.0/). The Open Database License (ODbL) is a license agreement intended to allow users to freely share, modify, and use this Dataset while maintaining this same freedom for others, provided that the original source and author(s) are credited.Nyaton Kitnya, M.V. Prabhudev, Chet Prasad Bhatta, Thai Hong Pham, Tshering Nidup, Karsing Megu, Jharna Chakravorty, Axel Brockmann, Gard Otis
